# The Effects of Programmed Cell Death of Mesenchymal Stem Cells on the Development of Liver Fibrosis

**DOI:** 10.1155/2023/4586398

**Published:** 2023-05-11

**Authors:** Hong-wei Wu, He-dan Chen, Ya-hong Chen, Xin-li Mao, Yu-yi Feng, Shao-wei Li, Xian-bin Zhou

**Affiliations:** ^1^Department of Infectious Diseases, Taizhou Enze Medical Center (Group) Enze Hospital, Taizhou, Zhejiang, China; ^2^Department of Infectious Diseases, Taizhou Hospital of Zhejiang Province Affiliated to Wenzhou Medical University, Linhai, Zhejiang, China; ^3^Health Management Center, Taizhou Hospital of Zhejiang Province Affiliated to Wenzhou Medical University, Linhai, Zhejiang, China; ^4^Department of Gastroenterology, Taizhou Hospital of Zhejiang Province Affiliated to Wenzhou Medical University, Linhai, Zhejiang, China; ^5^Key Laboratory of Minimally Invasive Techniques & Rapid Rehabilitation of Digestive System Tumor of Zhejiang Province, Taizhou Hospital Affiliated to Wenzhou Medical University, Linhai, Zhejiang, China; ^6^Institute of Digestive Disease, Taizhou Hospital of Zhejiang Province Affiliated to Wenzhou Medical University, Linhai, Zhejiang, China

## Abstract

Mesenchymal stem cells have shown noticeable potential for unlimited self-renewal. They can differentiate into specific somatic cells, integrate into target tissues via cell-cell contact, paracrine effects, exosomes, and other processes and then regulate the target cells and tissues. Studies have demonstrated that transplantation of MSCs could decrease the expression and concentration of collagen in the liver, thereby reducing liver fibrosis. A growing body of evidence indicates that apoptotic MSCs could inhibit harmful immune responses and reduce inflammatory responses more effectively than viable MSCs. Accumulating evidence suggests that mitochondrial transfer from MSCs is a novel strategy for the regeneration of various damaged cells via the rescue of their respiratory activities. This study is aimed at reviewing the functions of MSCs and the related roles of the programmed cell death of MSCs, including autophagy, apoptosis, pyroptosis, and ferroptosis, as well as the regulatory pathogenic mechanisms of MSCs in liver fibrosis. Research has demonstrated that the miR-200B-3p gene is differentially expressed gene between LF and normal liver samples, and that the miR-200B-3p gene expression is positively correlated with the degree of liver fibrosis, suggesting that MSCs could inhibit liver fibrosis through pyroptosis. It was confirmed that circulating monocytes could deliver MSC-derived immunomodulatory molecules to different sites by phagocytosis of apoptotic MSCs, thereby achieving systemic immunosuppression. Accordingly, it was suggested that characterization of the programmed cell death-mediated immunomodulatory signaling pathways in MSCs should be a focus of research.

## 1. Introduction

Mesenchymal stem cells (MSCs), a group of unique cells, have shown great potential for unlimited self-renewal [1]. They are found in various tissues, including the liver, bone marrow, intestine, connective tissues, spleen, and placenta [2, 3]. MSCs can not only differentiate into mesenchymal cell lines (e.g., osteocytes, chondrocytes, skeletal muscle cells) but also develop into ectodermal and endodermal cells (e.g., hepatocytes and neurons) [4–6]. MSCs are becoming a focus of research in the field of cell therapy due to their unique properties, including easy acquisition, specific recruitment to the damaged site, and low ethical restrictions [7]. In addition, one feature of MSCs is the lack of costimulatory molecules, including CD80, CD86, and HLA-II, which could result in the failure of MSCs to induce an immune response [8]. To date, stem cell technology has undergone great progress, and it may be used to treat various diseases related to the nerves, lung, heart, and liver [9]. Studies have demonstrated that through cell-cell contact, paracrine effects, and exosomes, MSCs may differentiate into cardiomyocyte-like cells, integrate into host tissue, and enhance resident cell activity [10]. MSC-mediated immunomodulation functions through the synergy of cell contact-dependent mechanisms and soluble factors. Hence, the potential application of MSCs as therapeutic agents for autoimmune and inflammatory diseases was confirmed [11].

Current bottlenecks in the therapeutic use of MSCs include donor-to-donor variability and the need for ex vivo expansion. The phenotype and function of MSCs are affected by the donor's age, body mass index (BMI), lifestyle, and pathophysiological conditions, leading to significant heterogeneity in MSCs isolated from different donors [12, 13]. The extended ex vivo expansion of isolated MSCs can further affect their clonogenicity, proliferative potential, and functionality. The therapeutic potential of MSCs is mainly attributed to two aspects: first, the replacement of the damaged tissue by differentiating into various cell lineages; and second, the regulation of immune responses by the immunomodulatory function. The major mechanism underlying MSC-based therapy is the paracrine function, which secretes various soluble factors to exert immunomodulatory, angiogenic, antiapoptotic, and antioxidative effects [14]. Induced pluripotent stem cell- (iPSC-) derived MSCs can be synthesized using a new cell differentiation and expansion platform that eliminates major issues of supply, scalability, and consistency. The iPSC-based approach has the potential to overcome the fundamental limitations of conventional, donor-derived MSC production processes as it facilitates the synthesis of an effectively limitless number of MSCs from a single blood donation. In addition, the need for excessive culture expansion of differentiated MSCs can be avoided by harnessing the indefinite replication potential of iPSCs [15].

Liver fibrosis (LF), which is regulated by activated hepatic stellate cells (HSCs), is a common cause of various chronic liver diseases. After liver injury, HSCs undergo phenotypic transformation from resting HSCs to myofibroblast-like cells, which may stimulate the expression of *α*-smooth muscle actin (*α*-SMA) and promote the synthesis of extracellular matrix (ECM) [16]. Endogenous tissue inhibitors of metalloproteinases (TIMPs) reduce excessive proteolytic ECM degradation by matrix metalloproteinases (MMPs), and an imbalance in the MMPs/TIMPs activity ratio may underlie the pathogenesis of LF. Therefore, the crucial objective of LF treatment is to inhibit the production of ECM and degrade its components. Cytokine transforming growth factor-*β* (TGF-*β*) is considered an essential factor in the LF. Once binding with the receptor, TGF-*β* triggers the activation of a signaling cascade, promoting the proliferation of prefibrotic cells and myofibroblasts. Therefore, the TGF-*β* pathway is critical for antifibrosis therapy, and the regulatory mechanisms have been extensively investigated in different preclinical and clinical trials [17].

The development and homeostasis of multicellular organisms are not only associated with the regulation of cell proliferation but also with the disposal of damaged cells. Programmed cell death can be induced by developmental programs and stress-induced signals, stimulating membrane-bound, and cytosolic proteins that trigger cell death via intricate cascades of transcriptional changes and posttranslational protein modifications [18]. To date, mitochondrial transfer from MSCs has demonstrated protective effects on lung injury, bronchial epithelial injury, allergic diseases, damaged cardiomyocytes, alkali-burnt corneal epithelial cells, kidney injury, ischemic damage, neurotoxicity, and spinal cord injury. In addition, several signals were identified, including the release of damaged mitochondria, mitochondrial DNA, and mitochondrial products along with elevated levels of reactive oxygen species, triggering mitochondrial transfer from MSCs to the recipient cells [19, 20].

Therefore, the present study is aimed at assessing the influences of MSCs on LF and their potential clinical value, especially the mechanisms of programmed cell death of MSCs in LF through apoptosis, autophagy, pyroptosis, and ferroptosis. The findings may facilitate the understanding of the programmed cell death of MSCs and suggest a potential therapeutic method for LF.

### 1.1. Progress of Mesenchymal Stem Cells in LF Treatment

**Figure 1:**
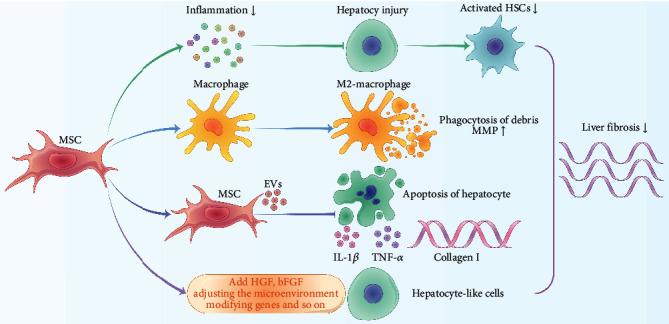
Mechanism of MSCs in reducing liver fibrosis. MSCs could increase the expression levels of anti-inflammatory cytokines (interleukin-10 (IL-10) and tumor necrosis factor-*α* (TNF-*α*)) and reduce the expression levels of proinflammatory cytokines (IL-6, IL-1A, IL-17, monocyte chemoattractant protein-1 (MCP-1), granulocyte colony-stimulating factor (GCSF), interferon-gamma (IFN-*γ*), macrophage inflammatory protein-2A (MIP-2A), and granulocyte-macrophage colony-stimulating factor (GMCSF)), thereby reducing hepatocyte injury and inhibiting the activation of hepatic stellate cells. MSCs enhance the generation of anti-inflammatory phenotypes in macrophages (M2 macrophages), phagocytic debris, and increase the production of MMP to reduce the degree of liver fibrosis. MSC-EVs inhibit the production of TNF-*α*, IL-1*β*, and collagen-1*α*, and thus reduce hepatocyte apoptosis and inhibit liver fibrosis. The ability of MSCs to differentiate into hepatocyte-like cells can be improved by adding cytokines and growth factors, regulating the microenvironment, and modifying genes, which could relieve liver fibrosis.

Animal and clinical studies revealed that MSCs derived from bone marrow or other tissues might be transformed into hepatocyte-like cells cultured in specific conditions *in vitro* and show the normal metabolic functions of hepatocytes [21–23]. MSCs also contribute to the recovery of liver function to some extent and suppress inflammation in the liver, thereby exhibiting a potential therapeutic effect on LF [24]. Studies have indicated that in LF models, transplantation of MSCs could significantly reduce the expression of collagen and its concentration in the liver, which is associated with the reduced degree of fibrosis [25–28]. Sakaida et al. used fluorescent protein-labeled bone marrow-derived MSCs (BMSCs) to treat mice with LF induced by carbon tetrachloride (CC1_4_), and the results showed that LF was alleviated in mice that underwent transplantation of MSCs and that their survival rate was significantly improved [29]. A meta-analysis evaluated the efficacy and the safety of BMSCs in decompensated cirrhosis, and it was revealed that, at 8, 16, 24, and 48 weeks after the infusion of MSCs, the albumin level was significantly elevated, and the end-stage liver disease score, alanine aminotransferase level, total bilirubin level, and prothrombin time improved to different degrees with no serious adverse events or complications [30]. Kharaziha et al. conducted a phase I-II clinical trial of autologous BMSCs to treat LF with multiple causes, and it was demonstrated that MSCs could improve liver function [31]. Jang et al. and Suk et al. found that autologous BMSC transplantation safely improved histologic fibrosis and liver function in patients with alcoholic cirrhosis [32, 33]. MSCs suppress the proliferation of HSCs and the *α*-SMA expression level via cell-to-cell contact, where the Notch signaling pathway possibly plays a crucial role [34, 35]. It was also confirmed that transplantation of MSCs promoted the activation of MMP-9 and MMP-13, while it weakened the activation of TIMP-1, thereby promoting the degradation of collagen and other ECM proteins in LF [27, 34, 36]. It has also been found that MSCs play an antifibrosis role via extracellular vesicles (EVs) or exosomes. EVs from human umbilical cord-derived MSCs were used to treat mice with LF induced by CCl_4_ and were found to slow the progression of LF, alleviate liver inflammation, and reduce collagen deposition. A previous study indicated that this process may be realized through dysfunction of the TGF-*β*1/SMAD signaling pathway [37]. In a liver injury study, EVs produced by MSCs showed a protective effect on liver cells in drug-induced liver injury models, suggesting that MSCs may partially restore the liver function by upregulating liver cell proliferation [38]. In a thioacetamide- (TAA-) induced rat model of LF, EVs from human embryonic stem cell- (ESC-) derived MSCs could reduce the degree of LF. A gene expression analysis also showed that after rats with LF were treated by MSC-EVs and MSCs, the expression levels of collagenases (e.g., MMP13 and MMP9), anti-inflammatory cytokines (e.g., IL-10 and TGF-*β*1), and antiapoptotic genes (e.g., BCL-2) were all upregulated, while the expression levels of proapoptotic genes (BAX), proinflammatory cytokines (e.g., TNF-*α*, IL-2), and the main factors contributing to fibrosis (Coll*α*, *α*-SMA, and TIMP1) were downregulated. It was suggested that MSC-EVs could regulate the hepatic inflammatory microenvironment through the downregulation of immune cell infiltration and the regulation of both the expression and secretion of inflammatory factors, including anti-inflammatory and proinflammatory factors [3, 39]. Other EVs from various sources of MSCs have shown similar effects on liver protection and regeneration, including BMSCs [40–42], human menstrual blood-derived MSCs [43], adipose-derived MSCs, and human liver-derived MSCs [44, 45] (Figure 1).

### 1.2. Programmed Cell Death of Mesenchymal Stem Cells

#### 1.2.1. Apoptotic MSCs

For a long time, it was assumed that different functional MSCs were efficacious for disease treatment. However, recent studies have suggested that the survival role of MSCs may be affected, and apoptotic MSCs (apo-MSCs) may also have a protective effect on the inflammatory microenvironment *in vivo* [46–48].

Most intravenously injected MSCs may be trapped in the capillary beds in the lung [49]. However, in the respiratory system, the total number of viable MSCs can rapidly decrease within one day [49]. Almost all captured MSCs are phagocytosed by alveolar macrophages, circulating neutrophils, and monocytes and are then redistributed throughout the system, accumulating primarily in the liver and spleen [11]. Although pulmonary transplantation of MSCs has emerged as a leading clinical intervention, several studies suggested that direct injection into the damaged tissue exhibited outcomes similar to intravenous injection, and the apoptosis of locally transplanted MSCs was reported after 3-5 days. Within one week, nearly all MSCs that were locally transplanted appeared in tissue-specific phagocytes [11]. It was revealed that the immune regulation mediated by MSCs can be achieved through apoptotic, metabolically dysfunctional, or fragmented MSCs. There is also evidence that apo-MSCs can inhibit harmful immune responses and reduce persistent inflammation more effectively than viable MSCs [11]. The dramatic decrease in the number of viable MSCs does not affect the immunosuppressive or therapeutic effects of MSCs, and apo-MSCs can regulate the role of immune cells [11].

Apoptosis plays a pivotal role in the arbitration of cell deletion in tissue homeostasis, embryological development, and immunological functioning [50–52]. Cells undergo a series of morphological changes, including cytoplasmic contraction and nuclear aggregation during the apoptosis of MSCs [50, 53–55]. Subsequently, the activated cysteine protease cleaves Rho-associated protein kinase 1 (ROCK1) to produce a truncated kinase with bioactivity of actin-myosin remodeling and cell contraction [56]. The membrane gradually protrudes, accompanied by blistering and fragmentation, eventually forming apoptotic fragments and apoptotic extracellular vesicles (apo-EVs) [57]. Apo-EVs have been demonstrated to precisely modulate the function of the immune system, such as T cells and macrophages, and improve tissue repair, including the regeneration of skin and protection for blood vessels [58–60]. A growing body of evidence has demonstrated that apo-EVs are key mediators of MSCs, and that the administration of apo-EVs is a promising cell-free therapeutic strategy [47, 61]. Apo-EVs play a regulatory role in a precisely tuned molecular network through phagocytosis or dynamic interaction with recipient cells [61, 62]. Notably, the direct delivery of apo-MSCs and apo-EVs is associated with excessively viable MSCs [63, 64]. Therefore, the transplantation of MSC-derived apo-EVs is expected to treat a number of diseases, including but not limited to osteoporosis, myocardial infarction, colitis, and graft-versus-host disease [54, 61, 64–66].

Galleu et al. utilized activated peripheral blood mononuclear cells (PBMCs) from healthy donors *in vitro* to determine the driving mechanism of apoptosis in MSCs. When PBMCs were activated, early apoptosis of MSCs was induced, which peaked within 4 h and shifted to late apoptosis within 24 h. According to the *in vivo* observations, the activation of caspase 3 in MSCs could only be induced by activated PBMCs, which peaked at 90 min, and this phenomenon was thoroughly eliminated by the pan-caspase inhibitor Z-VAD-FMK [47].

A study also showed that CD56+ NK and CD8+ T cells participated in the apoptosis of MSCs. To characterize the mechanism by which activated cytotoxic cells induce MSC apoptosis, the related factors involved in the activation of caspase 3 were investigated. The inhibition of granase B (GrB) or perforin completely eliminated the ability of activated PBMCs to kill MSCs or activate caspase 3. It was also revealed that CD95 ligands (CD95L or Fas ligands (Fas-L or apoptotic antigen 1 ligand)) were neutralized, while they were not neutralized after suppressing TNF-*α* or TNF-related apoptosis-inducing ligands [47]. The intrinsic phenomenon of the MSC-cytotoxic cell interaction was detected, and Galleu et al. found that apoptosis was unaffected by antihuman leukocyte antigen I (anti-HLA I)- or anti-HLA II-neutralizing antibodies. Activated PBMCs showed no difference in cytotoxic activity against autologous or allogeneic MSCs. Therefore, direct cell contact is important for the immune-suppressive effects of PBMCs to induce apoptosis and block immune synapse formation via the inhibition of polarization in the center of microtubule tissues. The results showed that there is a bystander effect when activated cytotoxic cells kill MSCs without the involvement of immunological synapses [47].

Apoptosis of MSCs increases the ability of MSCs to induce immunosuppressive phenotypes in macrophages, dendritic cells (DCs), and T cells. As apo-MSCs are more readily phagocytic than viable MSCs, CTL-dependent apoptosis of transplanted MSCs is a prerequisite for the MSC-based regulation of the phenotypes and roles of macrophages [67]. Under phagocytosis, apo-MSCs induce monocytes/macrophages to produce immunosuppressive macrophages (M2 macrophages) that promote the production of anti-inflammatory cytokines and growth factors, thereby mitigating inflammatory responses and enhancing the regeneration and recovery of damaged tissues. M2 macrophages suppress T cell proliferation in the liver in a PGE2- and IL-10-dependent manner, inhibit the production of inflammatory factors (IFN-*γ* and TNF-*α*) and profibrotic cytokines (TGF-*β*), and promote the proliferation of regulatory T (Treg) cells, thereby generating an immunosuppressive microenvironment in inflammatory tissues [68]. Notably, apo-MSC-mediated immune regulation is similar to or even superior to mediation by viable MSCs. The immunomodulatory effects of apo-MSCs are more predictable than those of viable MSCs [67].

### 1.3. Mesenchymal Stem Cell Autophagy and LF

As a cellular degradation pathway, autophagy utilizes lysosomes to get rid of damaged organelles or macromolecules, in which intracellular material circulation and an internal environmental balance can be achieved as amino acids are produced [69]. Based on substrate degradation and transportation methods, autophagy can be divided into three levels: macroautophagy, microautophagy, and chaperone-mediated autophagy [70]. Macroautophagy maintains cellular homeostasis through targeting cytoplasmic contents and organelles into autophagosomes for degradation [71]. In addition to basic homeostasis, autophagy is involved in the pathogenic mechanisms of a number of diseases, including cancer [72], inflammation [73], metabolic diseases [74], neurodegeneration [75], and cardiovascular diseases [76]. A number of studies have explored the functions of autophagy in particular diseases and also developed potential therapeutic strategies [77, 78]. MSC autophagy is critical for limiting inflammation, apoptosis, and oxidative stress in the cells associated with diseases, and it finally promotes treatment using MSCs [79]. It could modulate MSC-regulated immune regulation, suppress inflammation, and enhance anti-inflammation. In a previous study, MSCs were pretreated with 3-methyladenine (3-MA) and rapamycin to regulate autophagy; then, they were cocultured with CD4+ T cells, and it was found that 3-MA inhibited autophagy in MSCs, which was activated by rapamycin [80]. It was shown that rapamycin could increase the migration of CD4^+^ T cells, while treatment with 3-MA decreased their migration. Furthermore, MSC autophagy enhanced the migration of CD4^+^ T cells via CXCL8, promoted the differentiation of Treg cells, and inhibited the differentiation of type 1 T helper (Th1) cells via the secretion of TGF-*β*1. In addition, Gao et al. suggested that MSC autophagy could modulate the immunosuppression of CD4^+^ T cells through its influence on TGF-*β*1 secretion [81]. The homeostasis of CD4+ T cells was considered to be pivotal in hepatitis B virus-related LF [82, 83].

Wang et al. investigated the antifibrosis phenomenon of MSCs in the mouse model of CCl_4_-induced LF. MSCs were stimulated *in vitro* with fibrosis-related factors (TNF-*α*, interferon-*γ* (IFN-*γ*), and TGF-*β*1) to mimic the LF microenvironment. The results showed that MSCs responded to the LF microenvironment by upregulating the expression of beclin-1 (Becn1) and exhibiting autophagy *in vitro* and *in vivo*. After Becn1 was knocked out, MSC autophagy was inhibited, which enhanced the antifibrosis effect. The increased antifibrosis potential of MSCs may be attributed to the inhibition of T lymphocyte infiltration, hematopoietic stem cell proliferation, and the production of TGF-*β*1, IFN-*γ*, and TNF-*α* mediated by the paracrine PTGS2/PGE2 pathway [84]. However, tonsil-derived MSCs are differentiated into hepatocellular-like cells and inhibit LF by activating autophagy and downregulating TGF-*β* [85]. Thus, regulating MSC autophagy is a promising strategy for improving the antifibrosis function of MSCs.

### 1.4. Mesenchymal Stem Cell Ferroptosis and LF

As a type of iron-dependent programmed cell death, ferroptosis is driven by the intracellular peroxidation of lipids and induced by erastin [86], which is different from necrosis, apoptosis, and autophagy [86–89]. Morphologically, ferroptosis-induced cell death is characterized by mitochondrial contraction, increased membrane density, and mitochondrial crest reduction or disappearance. Ferroptosis does not show the morphology of typical phenotypes of apoptosis (i.e., chromatin aggregation and marginalization), necrosis (i.e., cytoplasm and organelle swelling or plasma membrane rupture), and autophagy (i.e., the formation of double-membrane vesicles) [86, 88–91]. Iron overload could affect ferritin deposition and systemic iron homeostasis [92, 93]. High concentrations of iron were found in the liver and spleen, leading to the aggregation and overproduction of ferritin-containing iron [94]. Ferritin covers light chain and heavy chain, including ferritin light chain (FTL) and ferritin heavy chain (FTH), which may act as a place for intracellular iron storage and play an essential role in the regulation of cell death [81, 95, 96]. The decreased levels of FTH1 and FTL in erastin-induced ferroptosis are correlated with the increased intracellular iron concentrations in MSCs. On the contrary, FTH1/FTL overexpression could alleviate ferroptosis in MSCs [97]. Bridle et al. demonstrated that HSCs express specific receptors for FTH and transferrin [98, 99]. FTH1 activates the expression levels of key profibrosis genes, including actin alpha-2 (ACTA-2), collagen-1, and bone morphogenetic protein-6 (BMP-6) by interacting with these receptors, which may activate HSCs and induce fibrosis [99, 100]. MSCs may inhibit LF by regulating ferroptosis through FTH1; however, further study is required to elucidate the specific mechanism.

### 1.5. Mesenchymal Stem Cell Pyroptosis and LF

**Figure 2:**
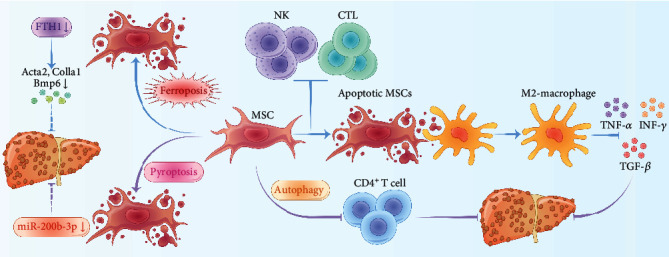
Programmed cell death of MSCs attenuates liver fibrosis. Autophagy of MSCs attenuates liver fibrosis through the immunosuppression of CD4^+^ T cells. NK cells and CTLs initiate apoptosis of MSCs, and apoptotic MSCs promote macrophages to produce an immunosuppressive (M2) phenotype and inhibit the production of TNF-*α*, TGF-*β*, and IFN-*γ*. Ferritin heavy chain-1 (FTH-1) activates the expression of key profibrotic genes, including Col1a1, bone morphogenetic protein-6 (BMP-6), and actin alpha-2 (ACTA-2), which may activate HSCs and induce liver fibrosis. The expression of FTH1 is low in iron-death MSCs, and iron-death MSCs may reduce liver fibrosis through the low expression of FTH1. miR-200b-3p is positively correlated with the degree of liver fibrosis, and the expression of miR-200b-3p decreases in the pyroptosis of MSCs, which may alleviate liver fibrosis.

Pyroptosis (inflammatory necrosis) is a type of inflammatory cell death that is mainly caused by microbial infection, accompanied by the activation of inflammasomes and maturation of proinflammatory cytokines. It plays an important role in the pathogenesis of several diseases [101–103]. Pyroptosis can be regulated by gasdermin-D (GSDMD) and caspase-1 [104, 105]. Inflammasomes, such as *NLR family CARD* domain-*containing protein 4* (NLRC4), absent in melanoma 2 (AIM2), *NLR* family *pyrin domain*-*containing 1b* (NlRP1b), and NLR family pyrin domain-containing 3 (NLRP3), activate caspase-1 by transforming procaspase1 into Cl-caspase1, and facilitate the formation of IL-18 and IL-1*β* from their precursors, leading to the mediation of pyroptosis. It was reported that pyroptosis is involved in the development of several inflammatory diseases [106]. Zhang et al. found that ORLNC1 regulates the BMSC pyroptosis induced by chronic myeloid leukemia through the miR-200b-3p/Foxo3 pathway *in vitro* and *in vivo*, and ORLNC1 is not correlated with the expression level of miR-200b-3p, which is critical for MSC pyroptosis [107]. Meanwhile, Ye et al. demonstrated that the miR-200B-3p gene is differentially expressed between LF and normal liver samples, and that its expression is positively correlated with the degree of LF [108], suggesting that MSCs can inhibit LF through pyroptosis, while further research is required (Figure 2).

### 1.6. Potential Pathways for Programmed Cell Death of Mesenchymal Stem Cells to Treat LF

It is broadly accepted that the indoleamine 2,3-dioxygenase (IDO)/kynurenine pathway is critical for the immunomodulatory properties of apo-MSCs. Apo-MSCs could increase immunosuppressive IDO in both macrophages and DCs [11]. Apo-MSCs injected intraperitoneally were phagocytosed by peritoneal CD11b+macrophages;however, this phenomenon was not observed in the macrophages from the lung or spleen [46]. In contrast, when apo-MSCs were administered intravenously, they were predominantly phagocytosed by CD11b^high^CD11c^int^, CD11b^high^CD11c^−^, and CD11b^−^CD11C^+^ phagocytes in the lung [46]. Importantly, apo-MSCs significantly enhanced the expression of IDO in macrophages and DCs and increased its immunosuppressive properties in an IDO-dependent manner [11].

IDO is a cytoplasmic and heme-containing enzyme that can convert tryptophan to quinolinic acid, which has an immunosuppressive function. The IDO1 enzyme catabolizes the conversion of L-Trp into KYN, which can be consequently processed enzymatically to several metabolites (i.e., KYNA, 3-hydroxykynurenine (3-HK), 3-hydroxyanthranilic acid, and finally to quinolinic and picolinic acids) [18]. The aggregation of 3-HAA, KYNA, QA, and 3-HK induced by IDO directly suppresses the activated T and B cells, resulting in a weakened adaptive immune response. In addition, kynurenine induces apoptosis of inflammatory cells in a Fas-independent manner by activating caspase-8 and releasing mitochondrial cytochrome C [18].

It is noteworthy that IDO is essential for the crossover between DCs and immunosuppressed Treg cells [18]. DCs promote the production and proliferation of Treg cells by increasing IDO activity, thereby inducing and maintaining immune tolerance. DCs induce the expression of lineage-defining transcription factor FoxP3 in naïve CD4^+^ T cells in an IDO/kynurenine-dependent manner, resulting in the production of CD4^+^ FoxP3^+^ Treg cells, which play an immunosuppressive role [18]. In the initial activation of T cell receptor- (TCR-) mediated resting Treg cells, protein kinase B (PKB/Akt) and mammalian target of rapamycin (mTOR) pathways may disrupt the immunomodulatory function of Treg cells and lead to the activation of a pro-inflammatory phenotype (pre-Tregs), producing more inflammatory cytokines. A limited tryptophan activation could regulate general control nonderepressible 2 (GCN2) kinase, thereby inhibiting Akt/mTOR2 signal transduction [18]. In order to prevent transdifferentiation of CD4^+^ T cells into Treg cells, DCs induce low tryptophan levels in an IDO/kynurenine-dependent manner and activate GCN2 kinase, thereby inhibiting the activation of the Akt/mTORC2 signaling pathway in Treg cells.

Similarly, the increased activation of GCN2 kinase and IDO in DCs could downregulate the TCR zeta chain of activated CD8^+^ CTLs, thereby reducing their cytotoxicity [18]. Consistent with these findings, apo-MSCs strengthen the immunosuppressive function and regulation of DCs and macrophages by increasing IDO activity [11]. Galleu et al. demonstrated that apo-MSCs successfully attenuated the macrophage-driven inflammatory response in mouse models by regulating the phagocytic activity and antigene presenting capabilities of DCs in an IDO/kynurenine-dependent manner [47]. IDO inhibition of apo-MSCs completely attenuated their anti-inflammatory effect, suggesting that IDO/kynurenine would be crucial for the apo-MSC-mediated regulation of phenotypes and the functions of macrophages and DCs [47]. In addition, circulating monocytes could deliver MSC-derived immunomodulatory molecules to different sites by phagocytosis of apo-MSCs, thereby achieving systemic immunosuppression [11]. Zhou et al. [85] administered MSCs to reduce the degree of LF, and they found that IDO inhibited the production of IL-17 by Th17 cells. Apo-MSCs may alleviate LF through the IDO/kynurenine pathway; however, further research is required to confirm this finding.

## 2. Conclusions

MSCs can ameliorate LF through suppressing the activation of HSCs or inhibiting the inflammatory microenvironment. Programmed cell death of MSCs inhibits harmful immune responses and reduces persistent inflammation more effectively than viable MSCs. Although some studies have provided evidence supporting the potential therapeutic application of MSC programmed cell death, the molecular mechanisms underlying their immunosuppressive regulation should be further clarified. Current bottlenecks in the clinical use of MSCs for therapy include donor-to-donor variability and the need for ex vivo expansion. The phenotype and function of MSCs are affected by the donor's age, BMI, lifestyle, and pathophysiological conditions, leading to significant heterogeneity in MSCs isolated from different donors. The extended *ex vivo* expansion of isolated MSCs can further affect their clonogenicity, proliferative potential, and functionality. Therefore, the characterization of programmed cell death-mediated immunomodulatory signaling pathways in MSCs should be a focus of research. In order to improve clinical safety, efficacy, and reliability, additional studies are needed to validate these effects on different stages of LF and explore etiologies.

## Data Availability

The data used to support the findings of this study are included within the article.

## Abbreviations

MSCs: Mesenchymal stem cells
PGE2: Prostaglandin E2
TGF-*β*: Transforming growth factor-*β*
IDO: Indoleamine 2, 3-dioxygenase
IL-10: Interleukin-10
IL-1RA: IL-1 receptor antagonist
HGF: Hepatic growth factor
NO: Nitric oxide
HO-1: Heme oxygenase-1
TNF-*α*: Tumor necrosis factor-*α*
TSG-6: TNF*α*-stimulated gene-6
DCs: Dendritic cells
NK: Natural killer cells
CTLs: Cytotoxic T lymphocytes
iPSC: Induced pluripotent stem cells
HSCs: Hepatic stellate cells
*α*-SMA: *α*-Smooth muscle actin
TIMPs: Tissue inhibitors of metalloproteinases
MMPs: Matrix metalloproteinases
ECM: Extracellular matrix
LF: Liver fibrosis
CC1_4_: Carbon tetrachloride
BMSCs: Bone marrow-derived MSCs
EVs: Extracellular vesicles
ROCK1: Rho-associated protein kinase 1
apo-EVs: Apoptotic extracellular vesicles
PBMCs: Peripheral blood mononuclear cells
anti-HLA I: Antihuman leukocyte antigen I
apo-MSCs: Apoptotic mesenchymal stem cells.
